# Utility of hepatic vein waveform and transient elastography in patients with Budd–Chiari syndrome who require angioplasty: Two case reports

**DOI:** 10.1097/MD.0000000000017877

**Published:** 2019-11-11

**Authors:** Takuma Nakatsuka, Yoko Soroida, Hayato Nakagawa, Naoki Okura, Jiro Sato, Masaaki Akahane, Masaya Sato, Yutaka Yatomi, Osamu Abe, Ryosuke Tateishi, Kazuhiko Koike

**Affiliations:** a Department of Gastroenterology, Graduate School of Medicine, The University of Tokyo, Bunkyo-ku, Tokyo, Japan; b Department of Clinical Laboratory Medicine, Graduate School of Medicine, The University of Tokyo, Bunkyo-ku, Tokyo, Japan; c Department of Radiology, International University of Health and Welfare, School of Medicine, Minato-Ku; d Department of Radiology, Graduate School of Medicine, The University of Tokyo, Bunkyo-ku, Tokyo, Japan.

**Keywords:** Budd–Chiari syndrome, congestive hepatopathy, elastography, hepatic vein waveform

## Abstract

**Rationale:**

Budd–Chiari syndrome (BCS), which causes congestive hepatopathy and aggravates cirrhosis, is typically treated by interventional angioplasty to ameliorate blood flow. X-ray venography is useful for the evaluation of inferior vena cava (IVC) stenosis and determination of treatment timing, but it is invasive and thus unsuitable for repeated examinations. The development of a simple method for the prediction of IVC stenosis would reduce the burden on patients with BCS.

**Patient concerns:**

We report here our experience of 2 patients with BCS who underwent percutaneous transluminal angioplasty (PTA). The first patient was a 39-year-old male who underwent PTA to expand his stenotic IVC. The second patient was a 19-year-old male who underwent PTA 3 times due to restenosis of his IVC.

**Diagnoses:**

Both patients were diagnosed with BCS with severe obstruction of the IVC.

**Interventions:**

We evaluated the hepatic vein (HV) waveform by Doppler ultrasonography and measured liver stiffness (LS) using transient elastography (TE) before and after PTA.

**Outcomes:**

In case 1, the phasic oscillation of the HV waveform recovered and the LS value decreased after PTA. Both improvements were maintained for ∼3 years, reflecting the long-term patency of the IVC. In case 2, the HV waveform and the LS value improved temporarily after PTA, but then deteriorated gradually. Monitoring of the HV waveform and LS value allowed retreatment prior to total occlusion of the IVC and abrogated the risk of intravascular needle puncture.

**Lessons:**

Monitoring of the HV waveform and the LS value enables safe management of patients with BCS who may require PTA.

## 1 Introduction

Budd–Chiari syndrome (BCS) is a rare clinical disorder caused by obstruction of the hepatic venous outflow tract, and can result in congestive hepatopathy. The resulting long-term liver congestion triggers hepatic fibrosis, leading to cirrhosis and hepatocellular carcinoma.^[1–3]^ In Asia, membranous obstruction of the inferior vena cava (IVC) is the major cause of BCS.^[4]^ Percutaneous transluminal angioplasty (PTA) is frequently performed to alleviate hepatic congestion in patients with BCS and membranous obstruction.^[5,6]^ However, restenosis of the IVC often occurs, even after successful treatment^[7]^; in cases of severe occlusion of the IVC, retreatment by intravascular needle puncture—an invasive procedure with the potential for severe complications—is required. Therefore, the periodic monitoring of IVC patency and determination of the appropriate timing of PTA prior to the development of severe IVC obstruction are mandatory. Catheter venography is the most direct method for the assessment of IVC patency, but it is unsuitable for repeated examinations due to its invasiveness. Doppler waveform of the hepatic vein (HV) revealed by abdominal ultrasonography (US) is classified into triphasic, biphasic, and monophasic patterns and is known to reflect several conditions and hemodynamics of the liver.^[8]^ Liver stiffness (LS) measured by elastography, which is a useful device for evaluation of liver fibrosis, also reflects the degree of liver congestion.^[9,10]^ In this report, we present the cases of 2 patients with BCS treated by PTA, in whom the combination of HV waveform assessment by Doppler US and LS measurement by transient elastography (TE) facilitated evaluation of the therapeutic effect and monitoring for postoperative restenosis.

## 2 Case presentations

### 2.1 Case 1

**Figure 1:**
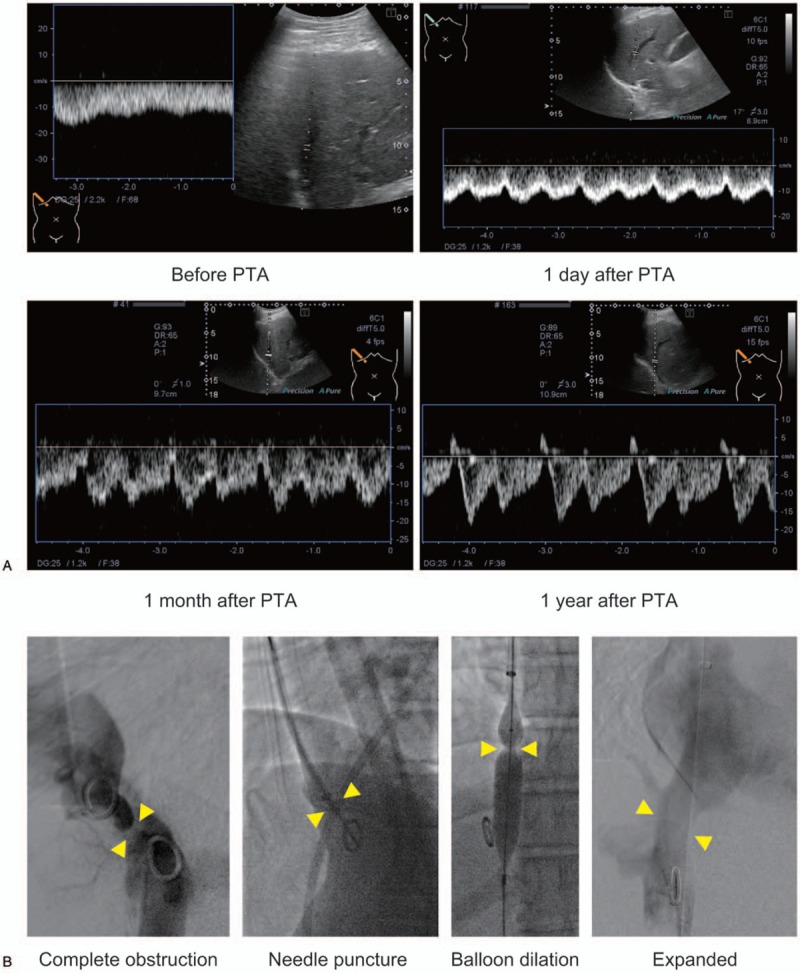
HV waveform before and after PTA in case 1. (A) The waveform of the RHV before and after PTA. The waveform was monophasic before treatment. One day after PTA, the HV waveform changed to biphasic and remained so for 1 month. One year after PTA, the HV waveform had adopted a normal triphasic pattern. (B) X-ray venography revealed complete obstruction of the IVC; thus, it was expanded by 14-gauge needle puncture followed by balloon dilation. After PTA, the contrast agent flowed from the IVC to the right atrium. (R)HV = (right) hepatic vein, IVC = inferior vena cava, PTA = percutaneous transluminal angiography.

A 39-year-old male with a history of a sustained low platelet count detected during health checkups presented to our hospital. US performed at an outpatient clinic had suggested the presence of chronic liver disease with splenomegaly and collateral formation of the portal vein. Contrast–enhanced computed tomography (CT) revealed severe stenosis of the IVC; based on this finding, the patient was diagnosed with BCS. We obtained HV waveforms using pulsed-wave Doppler devices as previously reported.^[11]^ The Doppler gate was placed on the right HV 1 to 3 cm distal to the inferior vena cava by an intercostal approach and a flattened monophasic pattern was detected that likely reflected blocked transmission of cardiac pulsation from the right atrium to the HV due to IVC stenosis (Fig. 1A). In addition, TE (FibroScan) yielded an LS value of 17.8 kPa, suggesting congestion and/or severe fibrosis of the liver.

X-ray venography showed complete obstruction of the IVC, and the pressures below and above the obstruction were 17 and 8 mm Hg, respectively (pressure gradient, 9 mm Hg; Fig. 1B). The IVC obstruction was treated by intravascular needle puncture followed by balloon dilation. This treatment decreased the pressure gradient between below and above the obstructed site to 2 mm Hg, indicating successful removal of the IVC obstruction.

On the day after PTA, the waveform of the patient's RHV, assessed by Doppler US, had changed to a biphasic pattern (Fig. 1A). His LS value decreased rapidly to 8.7 kPa, suggesting that the high LS value before PTA was due to congestion of the liver, rather than to liver fibrosis. One year after PTA, the patient's HV waveform had further improved to a triphasic pattern (Fig. 1A), and his LS value was 9.4 kPa. Three years after PTA, IVC restenosis had not occurred, the patient's HV waveform remained triphasic, and his LS value had decreased slightly to 7.2 kPa.

### 2.2 Case 2

An 11-year-old male was diagnosed with BCS at a pediatric hospital due to abnormal results of liver function tests. He had been followed at that hospital without interventional therapy, but 2 years after the diagnosis of BCS he presented to our hospital with severe edema in the legs and eyelids. Magnetic resonance imaging (MRI) revealed severe stenosis of the IVC due to a membranous structure. X-ray venography showed complete obstruction of the IVC; thus, intravascular needle puncture followed by balloon dilation was performed. After PTA, the diameter of the IVC had increased to 15 mm and the RHV flow had changed from retrograde to normal antegrade. The patient subsequently underwent routine follow-up at the pediatric hospital where he had been diagnosed with BCS.

**Figure 2:**
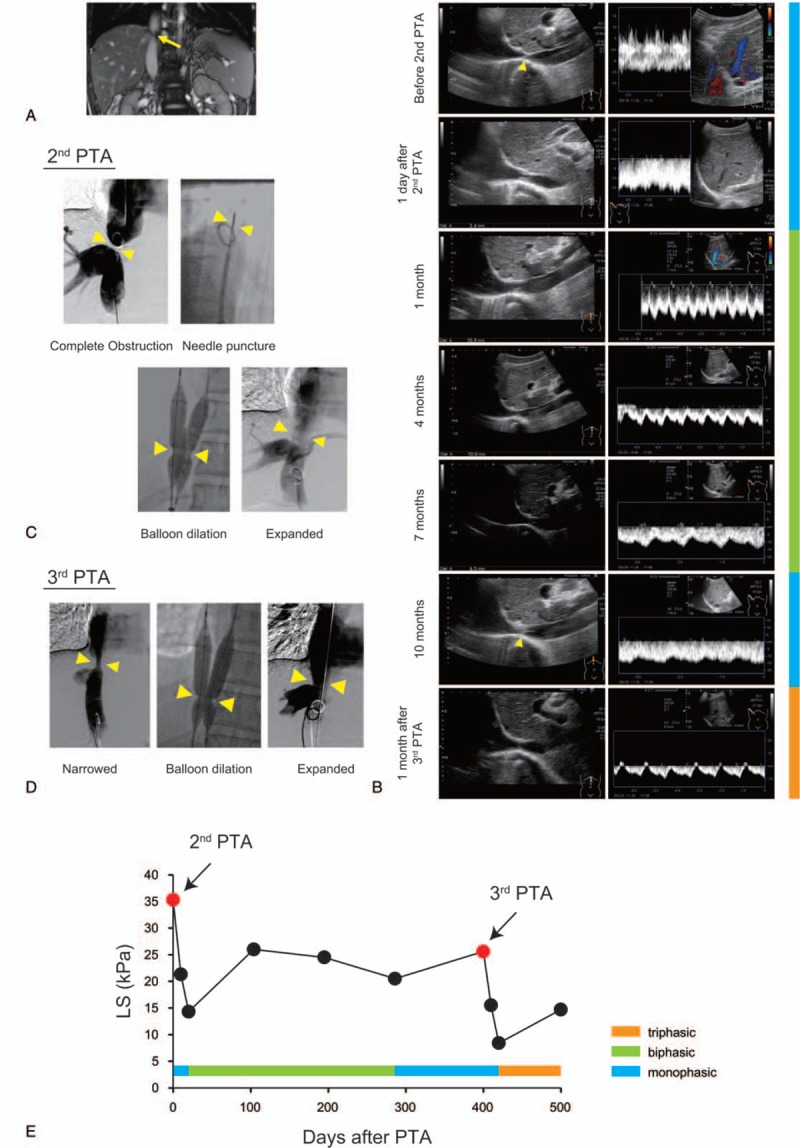
Changes in the HV waveform and LS value in case 2. (A) Enhanced MRI showed severe stenosis of the IVC (arrow). (B) Ultrasound images of the IVC and waveform of the RHV before and after PTA. Before the second PTA, a membranous structure was detected in the IVC (arrow) and the HV waveform was monophasic. One day after PTA, the IVC had opened slightly while the HV waveform remained monophasic. One month later, the IVC had expanded to 11 mm and a biphasic waveform was detected, which was maintained for 7 months. Ten months after the second PTA, the IVC had narrowed to 4.7 mm, the HV waveform exhibited a monophasic pattern, and a highly echoic structure was present in the IVC (arrow). After the third PTA, the IVC had expanded to 12.6 mm and the HV waveform was triphasic. (C) X-ray venography during the second PTA. The completely occluded IVC was expanded by 14-gauge needle puncture followed by balloon dilation. (D) X-ray venography during the third PTA; the narrowed IVC was expanded by balloon dilation. (E) Changes in the LS value and HV waveform pattern between before and after PTA. (R)HV = (right) hepatic vein, IVC = inferior vena cava, LS = liver stiffness, MRI = magnetic resonance imaging, PTA = percutaneous transluminal angiography.

At the age of 19 years, the patient was admitted to our hospital due to the exacerbation of lower–leg edema accompanied by splenomegaly and thrombocytopenia. Because MRI suggested restenosis of the IVC (Fig. 2A), we examined the HV waveform by Doppler US and assessed LS by TE. As expected, the HV waveform exhibited a flattened monophasic pattern (Fig. 2B) and the LS value was very high (35.3 kPa). As X-ray venography revealed complete obstruction of the IVC, intravascular needle puncture was performed, as in the first PTA (Fig. 2C). The pressure gradient across the obstruction was reduced from 11 to 8 mm Hg. On the day after PTA, the HV waveform remained monophasic (Fig. 2B), but the LS value had decreased to 21.3 kPa. One month after PTA, the HV waveform had changed to a biphasic pattern (Fig. 2B) and the LS value had decreased further to 14.3 kPa. The site of IVC stenosis remained patent (Fig. 2B).

After the second PTA, we monitored the HV waveform and LS value at 3–month intervals to detect restenosis of the IVC before it progressed to complete obstruction. At 10 months after the second PTA, the waveform of the RHV had a monophasic pattern (Fig. 2B) and the LS value had increased to 20.5 kPa. Furthermore, the IVC had narrowed to 4.7 mm and contained a highly echoic structure (Fig. 2B). Based on these data, we suspected restenosis of the IVC and performed X-ray venography. Although IVC stenosis had recurred, complete obstruction had not yet developed. The stenosis was treated successfully by balloon dilation without needle puncture (Fig. 2D). One month after the third PTA, the HV waveform exhibited a triphasic pattern and the LS value was 8.4 kPa (Fig. 2B, E).

## 3 Discussion

In patients with BCS, PTA can improve hepatic congestion and prevent liver fibrosis and portal hypertension; however, restenosis often occurs after the procedure.^[7]^ Because complete obstruction of the IVC increases the risk of complications of catheter treatment, timely intervention is critical.

The HV waveform is correlated with the severity of several hepatic diseases. Several groups, including ours, have reported that the HV waveform is associated with liver fibrosis.^[11–14]^ Liver fibrosis increases parenchymal stiffness and reduces HV wall compliance, which results in flattening of the HV waveform as liver fibrosis progresses. The HV waveform is also a useful indicator of BCS.^[6,15,16]^ The loss of phasic oscillation in the HV waveform indicates that cardiac movement is not transmitted to the HV, suggesting obstruction of hepatic outflow. Recovery of phasic oscillation in the HV waveform can be used to assess the therapeutic effect of balloon angioplasty.^[17,18]^ TE is used routinely for the noninvasive assessment of liver fibrosis. Liver congestion leads to an increased LS value as measured by TE^[9,10]^; therefore, TE can be used to assess the effect of PTA on hepatic congestion in patients with BCS.^[19]^

We investigated the utility of the HV waveform and the LS value measured by TE for the evaluation of the therapeutic effect of PTA and monitoring of the clinical course after angioplasty. In case 1, the patency of the IVC was maintained for several years after PTA, as indicated by a triphasic HV waveform and a low LS value. In case 2, the LS value increased gradually after the second PTA while the HV waveform remained biphasic, indicating the progression of hepatic congestion due to the development of IVC stenosis. The HV waveform ultimately flattened, suggesting severe restenosis of the IVC. Therefore, LS may be highly sensitive for the detection of IVC restenosis, and the HV waveform may be useful for determination of the timing of interventional therapy.

The finding of HV waveform must be carefully interpreted, as it reflects both cardiac and hepatic physiology as well as simple heartbeat. We have previously shown that HV waveforms can be detected even in patients with right heart dysfunction under Fontan circulation, thus circulatory dynamics might not greatly influence the shape of the HV waveform.^[14]^ In BCS patients who have already had cirrhosis due to prolonged liver congestion, the HV waveform may not improve after PTA, because the phasic waveform is lost as liver fibrosis progresses.^[11]^

In conclusion, we report 2 cases of BCS in which the HV waveform and the LS value determined by TE enabled the identification of IVC stenosis. Monitoring of the HV waveform and the LS value may be valuable for follow-up of patients with BCS, although further confirmatory studies are needed.

## Author contributions

**Conceptualization:** Takuma Nakatsuka, Yoko Soroida, Hayato Nakagawa.

**Data curation:** Takuma Nakatsuka, Yoko Soroida.

**Investigation:** Takuma Nakatsuka, Yoko Soroida.

**Methodology:** Takuma Nakatsuka, Yoko Soroida.

**Resources:** Naoki Okura, Jiro Sato, Masaaki Akahane.

**Supervision:** Masaya Sato, Yutaka Yatomi, Osamu Abe, Kazuhiko Koike.

**Writing – original draft:** Takuma Nakatsuka, Yoko Soroida.

**Writing – review & editing:** Hayato Nakagawa, Ryosuke Tateishi, Kazuhiko Koike.

Takuma Nakatsuka orcid: 0000-0002-5727-5385.

## References

[1] Menon KV Shah V Kamath PS The Budd-Chiari syndrome. N Engl J Med 2004;350 6:578–85. 14762185 10.1056/NEJMra020282

[2] Darwish Murad S Plessier A Hernandez-Guerra M Fabris F Eapen CE Bahr MJ Etiology, management, and outcome of the Budd-Chiari syndrome. Ann Intern Med 2009;151 3:167–75. 19652186 10.7326/0003-4819-151-3-200908040-00004

[3] Janssen HL Garcia-Pagan JC Elias E Mentha G Hadengue A Valla DC Budd-Chiari syndrome: a review by an expert panel. J Hepatol 2003;38 3:364–71. 12586305 10.1016/s0168-8278(02)00434-8

[4] Kage M Arakawa M Kojiro M Okuda K Histopathology of membranous obstruction of the inferior vena cava in the Budd-Chiari syndrome. Gastroenterology 1992;102 6:2081–90. 1587428 10.1016/0016-5085(92)90336-w

[5] Fisher NC McCafferty I Dolapci M Wali M Buckels JA Olliff SP Managing Budd-Chiari syndrome: a retrospective review of percutaneous hepatic vein angioplasty and surgical shunting. Gut 1999;44 4:568–74. 10075967 10.1136/gut.44.4.568 PMC1727471

[6] Valla DC The diagnosis and management of the Budd-Chiari syndrome: consensus and controversies. Hepatology 2003;38 4:793–803. 14512865 10.1053/jhep.2003.50415

[7] Han G Qi X Zhang W He C Yin Z Wang J Percutaneous recanalization for Budd-Chiari syndrome: an 11-year retrospective study on patency and survival in 177 Chinese patients from a single center. Radiology 2013;266 2:657–67. 23143028 10.1148/radiol.12120856

[8] Scheinfeld MH Bilali A Koenigsberg M Understanding the spectral Doppler waveform of the hepatic veins in health and disease. Radiographics 2009;29 7:2081–98. 19926763 10.1148/rg.297095715

[9] Millonig G Friedrich S Adolf S Fonouni H Golriz M Mehrabi A Liver stiffness is directly influenced by central venous pressure. J Hepatol 2010;52 2:206–10. 20022130 10.1016/j.jhep.2009.11.018

[10] Colli A Pozzoni P Berzuini A Gerosa A Canovi C Molteni EE Decompensated chronic heart failure: increased liver stiffness measured by means of transient elastography. Radiology 2010;257 3:872–8. 20935077 10.1148/radiol.10100013

[11] Soroida Y Nakatsuka T Sato M Nakagawa H Tanaka M Yamauchi N A novel non-invasive method for predicting liver fibrosis by quantifying the hepatic vein waveform. Ultrasound Med Biol 2019;45 9:2363–71. 31303401 10.1016/j.ultrasmedbio.2019.05.028

[12] Bolondi L Li Bassi S Gaiani S Zironi G Benzi G Santi V Liver cirrhosis: changes of Doppler waveform of hepatic veins. Radiology 1991;178 2:513–6. 1987617 10.1148/radiology.178.2.1987617

[13] Kawanaka H Kinjo N Anegawa G Yoshida D Migoh S Konishi K Abnormality of the hepatic vein waveforms in cirrhotic patients with portal hypertension and its prognostic implications. J Gastroenterol Hepatol 2008;23 (7 Pt 2):e129–36. 17924952 10.1111/j.1440-1746.2007.05155.x

[14] Nakatsuka T Soroida Y Nakagawa H Shindo T Sato M Soma K Identification of liver fibrosis using the hepatic vein waveform in patients with Fontan circulation. Hepatol Res 2019;49 3:304–13. 30182424 10.1111/hepr.13248

[15] Hosoki T Kuroda C Tokunaga K Marukawa T Masuike M Kozuka T Hepatic venous outflow obstruction: evaluation with pulsed duplex sonography. Radiology 1989;170 (3 Pt 1):733–7. 2644659 10.1148/radiology.170.3.2644659

[16] Bolondi L Gaiani S Li Bassi S Zironi G Bonino F Brunetto M Diagnosis of Budd-Chiari syndrome by pulsed Doppler ultrasound. Gastroenterology 1991;100 (5 Pt 1):1324–31. 2013376

[17] Ohta M Hashizume M Tomikawa M Ueno K Tanoue K Sugimachi K Analysis of hepatic vein waveform by Doppler ultrasonography in 100 patients with portal hypertension. Am J Gastroenterol 1994;89 2:170–5. 8304297

[18] Hirooka K Hirooka M Kisaka Y Uehara T Hiasa Y Michitaka K Doppler waveform pattern changes in a patient with primary Budd-Chiari syndrome before and after angioplasty. Intern Med 2008;47 2:91–5. 18195497 10.2169/internalmedicine.47.0501

[19] Mukund A Pargewar SS Desai SN Rajesh S Sarin SK Changes in liver congestion in patients with Budd-Chiari syndrome following endovascular interventions: assessment with transient elastography. J Vasc Interv Radiol 2017;28 5:683–7. 28153486 10.1016/j.jvir.2016.11.091

